# HIV, syphilis, hepatitis B and C in key populations: results of a 10-year cross-sectional study, Southern Brazil

**DOI:** 10.31744/einstein_journal/2022AO6934

**Published:** 2022-06-01

**Authors:** Breno Gonçalves da Silva, Laura Holtman Ferreira, Clea Elisa Lopes Ribeiro, Sonia Mara Raboni

**Affiliations:** 1 Universidade Federal do Paraná Curitiba PR Brazil Universidade Federal do Paraná, Curitiba, PR, Brazil.; 2 Secretaria Municipal da Saúde de Curitiba Curitiba PR Brazil Secretaria Municipal da Saúde de Curitiba, Curitiba, PR, Brazil.

**Keywords:** Sexually transmitted diseases, HIV, Population groups, Syphilis, Homosexuality, male, Sexual and gender minorities, Drug users, Sex workers

## Abstract

**Objective:**

Although the development of prevention and treatment strategies for sexually transmitted infections in key groups has improved over the years, they still remain a challenge for health systems worldwide. In this context, the objective of this study is to assess the seroprevalence in the tested population, with an emphasis on key populations, aiming at identifying the participants’ profile and consequently the development of testing strategies.

**Methods:**

The present study analyzed the seroprevalence of HIV, syphilis, and hepatitis B and C, and the epidemiological profiles of key and general populations tested at a reference public health facility for sexually transmitted infections testing and counseling in the city of Curitiba, Southern Brazil. A cross-sectional study was conducted to report data from 2010 to 2019.

**Results:**

A total of 9,086 samples were positive across all samples tested, and yielded 3,633 (5%) for HIV, 4,978 (10%) for syphilis, 340 (1%) for hepatitis C virus (HCV), and 135 (<1%) for hepatitis B virus (HBV). Overall, most of the participants were men (79 to 87%), and predominantly white. For HIV and syphilis, the predominant age groups were 21-30 years old (48 and 50%), HBV 21-40 years old (31%), and HCV 41-60 years old (25%). A high seroprevalence of HIV and syphilis was observed in the investigated key populations with a higher frequency in sex workers, men who have sex with men, and transgender.

**Conclusion:**

The progressive increase in syphilis cases emphasizes the need for effective interventions to enhance adherence to the use of condoms, and to expand diagnosis and treatment for these key populations.

## INTRODUCTION

Currently, sexually transmitted infections (STIs) represent a major challenge for the health system due to the growing number of cases and their socioeconomic impact. According to the World Health Organization (WHO), STIs are reaching the milestone of 1 million new cases per day worldwide.^(1,2)^ A comparison between 2017 and 2018 showed that syphilis increased 11.3% in men and 30.4% in women.^(3)^ A study conducted in some Brazilian cities showed an increase in human immunodeficiency virus (HIV) prevalence among men who have sex with men (MSM), from 12.1% in 2016 to 18.4% in 2019.^(4)^

The main difficulties in facing STIs are the prevention and treatment strategies used in key groups with higher rates of exposure and transmission, such as sex workers, MSM, transgender people, intravenous drug users (IDU), and other marginalized individuals who generally have restricted access to prevention and treatment actions.^(5)^

Since 1996, antiretroviral (ARV) treatment has been distributed for free in Brazil by the Brazilian Public Healthcare System (SUS - *Sistema Único de Saúde*) to improve the quality of life of people living with HIV/AIDS (PLWHA).^(6)^ However, due to the effectiveness of this treatment, people are neglecting the use of barrier prevention methods indicated for those infected with any STIs, increasing the chances of transmission of other infectious agents.^(7,8)^ Over the years, many studies have reported this scenario, showing that the syphilis incidence, especially among PLWHA and MSM, has been expressively increasing.^(7-9)^

Guidance and Counseling Centers (CTA - *Centro de Testagem e Aconselhamento*) have performed diagnosis through rapid tests for HIV, syphilis, and other STIs as a strategy to increase key populations’ attendance in health services. In addition to offering orientation and health care services, the staff is trained to better communicate with these key populations to avoid eliciting any feelings of discomfort or insecurity from the patient in relation this kind of service.

Moreover, high prevalence rates have been found in key populations as for hepatitis B (HBV) and C (HCV), diseases that share a similar route of transmission as syphilis and HIV. A study conducted by European Union countries and the United States of America (USA) showed that hepatitis C is usually more prevalent in IDUs, whose positive rates range from 13.8% to 84.3%.^(10)^ The incidence rates of HBV do not vary in key populations as much as other STIs, but its prevalence in IDUs, similarly to HCV, ranges from 0.5% to 6.1%.^(11)^ Hepatitis B virus infection rates are 1.4% in MSM, 0.4% in sex workers, and 2.7% in transgender.^(6,11,12)^

According to data from the Brazilian Ministry of Health, in a period of 10 years, from 2007 to 2017, 230,547 cases of HIV were reported. The distribution was more concentrated in the Southeast and South regions of Brazil, which had 47.4% and 20.5% of cases, respectively.^(13)^

The situation in Brazil is similar to the worldwide prevalence, with a higher number of cases in key populations compared to the general population.^(2,3)^ A study that estimated the prevalence of HIV and syphilis in the population of sex workers in Brazil found a prevalence of 4.9% for HIV and 2.5% for syphilis, values much higher than the 0.4% reported for the general population.^(6)^

A study conducted in the state of Rio Grande do Sul, south of Brazil, with data from 1998 to 2017, pointed out that the prevalence rates of syphilis and HCV were around 3.4% and 1.6%.^(14,15)^ Another survey conducted in the South region, in the state of Paraná, showed that the prevalence of syphilis is higher in patients with STIs, MSM, and drug users, representing 26.1%, 15.2%, and 11.9% of the positive cases, respectively.^(10)^

Globally, several actions have been taken to stop the spread of HIV and other STIs. Their success is directly associated to the estimation of the prevalence rates of these infectious diseases and intervention measures based on these estimates that can contribute to controlling them. In light of this, the objective of the present study is to analyze the seroprevalence of HIV, syphilis, and hepatitis B and C, and their demographic and epidemiologic characteristics, especially in the key populations tested at a referral service center in the city of Curitiba, Southern Brazil.

## OBJECTIVE

Assess the prevalence of HIV, syphilis, and hepatitis B and C in the general population and key populations tested in a reference service center in Curitiba, Southern Brazil, between 2010 and 2019.

## METHODS

This is an observational, analytical, cross-sectional study to assess the prevalence of HIV, syphilis, HBV, and HCV in the general population and key populations (transgender, IDUs, sex workers, and MSM) between 2010 and 2019.

### Site description

Curitiba is a city located in the state of Paraná, Southern Brazil, with a population of 1,948,626 inhabitants, and a Human Development Index (HDI) of 0.823, according to the most recent census.^(5)^ Since 2002, Curitiba has decentralized testing to the city-wide system of basic health units, which has resources to request testing, viral load test, and CD4 count to monitor the clinical condition of the patients.^(16,17)^

Over the years, the CTA has established itself as one of the most important testing centers in the city. Its main target audience are the key populations of MSM, sex workers, transvestites, transgender, and IDUs. The center has a multidisciplinary team, aiming to promote actions to encourage testing and reduce stigma regarding STIs, especially for the most socioeconomically vulnerable groups.^(16-18)^

This study reviewed the data records of standard protocol from the CTA. All personal information on the patients was excluded before accessing the database to preserve patient anonymity. Patients whose data were incomplete or unavailable, who did not get tested for the diseases contemplated in this study, or who did not fit the populations evaluated were excluded.

The key populations analyzed individually were MSM, transgender people, IDUs, and sex workers. This study did not include patients who were classified as another type of key population (“illicit drug user”, “truck driver”, “person living with another HIV positive”, “STI carrier”, “hepatitis carrier”, “health professional”, “homeless”, and “person in social exclusion”). Patients who did not classify as part of a key population were identified as part of the general population.

### Statistical analysis

Data were compiled in an Excel spreadsheet and statistical analysis was performed using the R 3.4.0 (R Core team, 2017). A descriptive analysis was conducted to depict the general characteristics of the population sample. Analytical analysis using statistical tools was applied, when appropriate, as continuous variables to describe mean or median values; categorical variables were described as numbers and frequencies. Qualitative variables were assessed using Fisher’s exact test; quantitative variables were compared using Student’s *t*-test. When several tests were considered using the Bonferroni adjustment, no significant difference in the primary results was observed. Independent analyses of subgroups were performed to determine the prevalence of STIs in the populations of interest. A value of p<0.05 was set as statistically significant.

The Research Ethics Committee of the Curitiba Municipal Health Department (SMS) approved this study (CAAE: 92987318.0.0000.0101, protocol: 2.812.493).

## RESULTS

**Figure 1:**
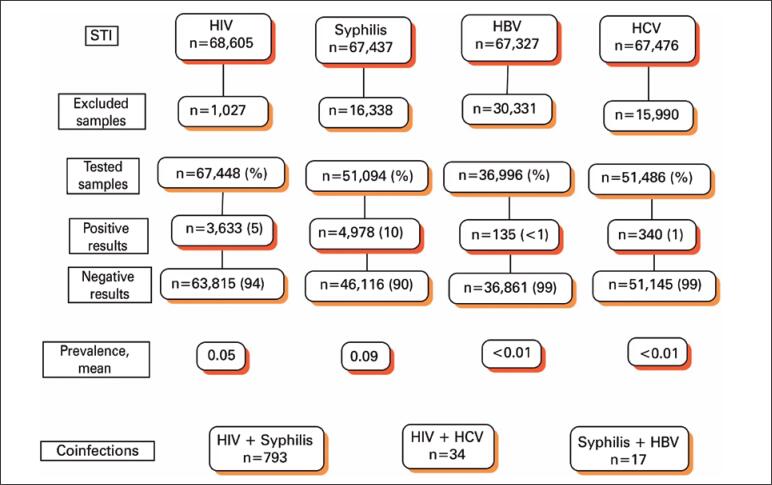
Flowchart summarizing the profile of the patients included in the study, Southern Brazil STI: sexually transmitted infections; HIV: human immunodeficiency virus; HBV: hepatitis B; HCV: hepatitis C.

During the study, of the 68,605 people that were assisted at the CTA, approximately 67,448 patients tested for HIV, among them 3,633 tested positive; 51,094 people tested for syphilis, of which 4,978 tested positive; 36,996 tested for HBV, among them, 135 tested positive, and finally, 51,486 patients tested for HCV, and of these, 340 tested positive. The study showed a total of 9,086 positive tests. Regarding co-infections, 793 (0.16%) tested positive for HIV and syphilis, 34 (0.02%) for HIV and HCV, and 17 (0.03%) for syphilis and HBV (Figure 1).

### Clinical-epidemiologic characteristics

Regarding HIV, syphilis, and HBV, the group with positive results was predominantly men (79 to 87%), white (70 to 73%), and in the age group from 21 to 30 years old (48 and 50%). For HCV, most people who tested positive were aged from 41 to 60 (25%).

The key population represented most of HIV and syphilis positive cases (62 to 63%), while HBV and HCV occurred more frequently in the general population (67 to 81%) (Table 1).

**Table 1:** Demographic and epidemiological data of positive cases

Categories		HIV	Syphilis	Hepatitis B	Hepatitis C
Positive samples, n (%)		3,633	4,978	135	340
Male, n (%)		3,095 (85)	4,333 (87)	114 (84)	268 (79)
Ethnicity, n (%)					
	White	2,540 (70)	3,549 (71)	95 (70)	249 (73)
	Not white	1,053 (29)	1,345 (27)	39 (29)	86 (25)
	Not informed	40 (1)	84 (2)	1 (1)	5 (2)
Median age (IQR) - years		29 (24-37)	29 (24-37)	36 (29-46)	44 (32-52)
Age group, years, n (%)							
	0-10	2 (1)	3 (0)	0	0
	11-20	280 (8)	378 (8)	0	15 (4.4)
	21-30	1,740 (48)	2,458 (50)	41 (30.4)	56 (16.4)
	31-40	953 (26)	1,164 (23)	42 (31)	74 (21.7)
	41-50	428 (11)	551 (11)	31 (23)	87 (25.8)
	51-60	182 (5)	275 (5)	20 (15)	85 (25)
	61 years old or more	47 (1)	147 (3)	1 (0.7)	23 (6.7)
	Not informed	1 (0)	2 (0)	0	0
Education level, n (%)					
	Elementary school	521 (14.3)	518 (10.4)	33 (24.4)	76 (22.3)
	High school	1,630 (44.9)	2,146 (43.1)	56 (41.5)	147 (43.2)
	University education	1,362 (37.5)	2,035 (40.8)	42 (31.1)	85 (25)
	None	6 (0.2)	8 (0.2)	0	5 (1.4)
	Not informed	114 (3.1)	271 (5.4)	4 (3)	27 (7.9)
Single, n (%)					
	Yes	2,867 (79)	4,253 (85)	98 (73)	238 (70)
	No	691 (19)	138 (3)	34 (25)	94 (28)
	Not informed	75 (2)	587 (12)	3 (2)	8 (2)
General population, n (%)		1,365 (37.6)	1,842 (37)	91 (67)	277 (81)
Key population, n (%)		2,248 (61.9)	3,136 (63)	44 (32.6)	63 (18.5)
	Men who have sex with men	2,165 (60)	3,007 (60.4)	40 (29.6)	57 (16.7)
	Sex workers	26 (0.70)	50 (1)	1 (0.7)	2 (0.5)
	Transgender people	46 (1)	74 (1.5)	3 (2.2)	2 (0.5)
	Injection drug user	11 (0.30)	5 (0.1)	0	2 (0.5)
Population not informed, n (%)		20 (0.55)	0	0	0

IQR: interquartile range; HIV: human immunodeficiency virus.

### HIV and syphilis infections

In nine years of collected data, 67,579 people were tested for HIV, of which 3,633 (5%) tested positive and 63,815 (94%) tested negative.

The profile of the HIV positive group tested at the CTA was white (70%), with an average age of 29 years old (IQR, 24-37), and single (79%) (Table 2). More than half of the patients belonged to the key group (62%), mainly MSM (60%), followed by transgender (1%) and sex workers (0.71%). Most people with negative results corresponded to the general population (70%).

**Table 2:** Demographic and epidemiological data of HIV and syphilis positive and negative cases

Categories		HIV			Syphilis		
		+	-	p value	+	-	p value
Samples, n (%)		3,633	63,815		4,978	46,116	
Men, n (%)		3,095 (85)	46,700 (73)	<0.0001	4,333 (87)	33,495 (72)	<0.0001
Ethnicity, n (%)							
	White	2,540 (70)	48,074 (75)	<0.0001	3,549 (71.4)	34,982 (76)	<0.0001
	Not white	1,053 (29)	14,940 (23)		1,345 (27)	10,506 (22)	
	Not informed	40 (1)	800 (1)		84 (1.6)	1,132 (2)	
Median age (IQR) - years		29 (24-37)	29 (24-38)	0.0014	29 (24-37)	29 (23-37)	0.0034
Age group, years, n (%)							
	0-10	2 (0)	39 (0)	<0.0001	3 (0.1)	27 (0)	<0.0001
	11-20	281 (8)	6,701 (10.5)		378 (7.5)	5,011 (10.9)	
	21-30	1,740 (48)	28,496 (44.6)		2,460 (49.4)	20,512 (44.5)	
	31-40	953 (26)	15,936 (25)		1,164 (23.3)	11,512 (24.9)	
	41-50	428 (12)	7,484 (11.7)		551 (11.1)	5,335 (11.6)	
	51-60	182 (5)	3,431 (5.4)		275 (5.5)	2,495 (5.4)	
	61 years old or more	47 (1)	1,728 (2.8)		147 (2.9)	1,224 (2.6)	
	Not informed	1 (0)	0		0	0	
Education level, n (%)							
	Elementary school	521 (14.3)	6,195 (10)	<0.0001	518 (10.4)	4,318 (9.3)	0.0004
	High school	1,630 (44.9)	25,433 (39.9)		2,146 (43.1)	18,065 (39.1)	
	University education	1,362 (37.5)	29,513 (46)		2,035 (40.8)	21,647 (46.9)	
	None	6 (0.2)	113 (0.1)		8 (0.16)	71 (0.1)	
	Not informed	114 (3.1)	2,561 (4)		271 (5.4)	2,015 (4.3)	
Single, n (%)							
	Yes	2,867 (79)	50,627 (79.3)	0.4027	4,253 (85)	36,730 (79.6)	<0.0001
	No	691 (19)	11,760 (18.4)		138 (3)	8,288 (17.9)	
	Not informed	75 (2)	1,428 (2.2)		587 (12)	1,098 (2.4)	
General population, n (%)		1,365 (37.6)	44,982 (70.5)	<0.0001	1,842 (37)	33,613 (73)	<0.0001
Key population, n (%)		2,248 (61.9)	18,711 (29.3)		3,136 (63)	12,495 (27)	
	Men who have sex with men	2,165 (60)	17,809 (28)		3,007 (60,5)	11,952 (25.9)	
	Sex workers	26 (0.7)	596 (0.90)		50 (1)	377 (0.8)	
	Transgender people	46 (1)	264 (0.41)		74 (1.4)	138 (0.3)	
	People who inject drugs	11 (0.2)	42 (0)		5 (0.1)	28 (0.1)	
Population not informed, n (%)		20 (0.5)	122 (0.2)		0	8 (0.01)	

IQR: interquartile range; HIV: human immunodeficiency virus.

**Figure 2:**
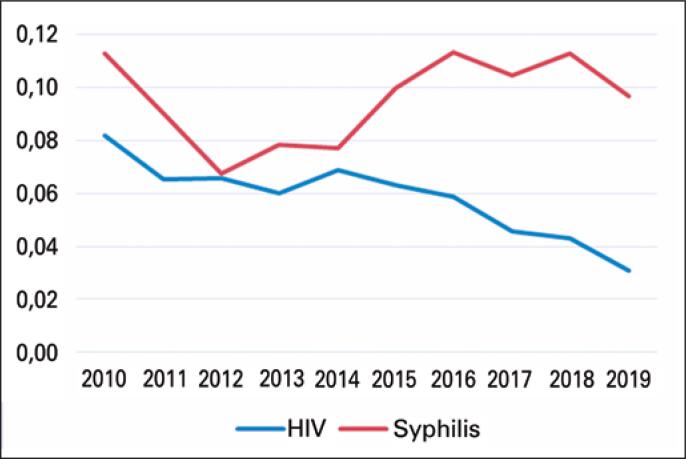
Syphilis and HIV seroprevalence rates from 2010 to 2019

Considering the distribution of HIV seroprevalence over the years, the reduction of positive cases is noticeable (from 8% to 2.5%), as shown in figure 2.

Regarding syphilis testing, more than half of the key population showed positive results (63%). Men who have sex with men (60%) were the majority of those, followed by sex workers (1%) and transgender (1%). As for people with negative results, a higher concentration of positive results was found among the general population (73%). The seroprevalence rates, as opposed to those of HIV, showed progressive increase after 2014, and have maintained levels above 10% since 2016 (Figure 2).

### Hepatitis B and C infections

A much smaller number of people were tested as for hepatitis: 36,996 people for HBV and 51,486 for HCV. The positivity rates were very low, 135 and 340 positive results were obtained for hepatitis B and C, respectively, with a seroprevalence mean below 1%.

In both of the tested groups, there was a predominance of men (84%), white (70 to 73%), aged between 29-52 years (Table 1), and most participants with positive results were identified as part of the general population (67 to 81%).

### General and key populations comparison

Both general and key populations presented a similar demographic profile, and the differences between those groups concern education level and mean age (Table 3). The study found a predominance of people aged 31 for HIV and 29 for syphilis, among those in the general population who tested positive for HIV or syphilis, whereas the key population showed a prevalence of people aged 27 (Table 3). As for education, the majority of the general population was composed of people who completed secondary education (46%), while the key population was composed of people who completed higher education (47.4%).

**Table 3:** Demographic and epidemiological data of HIV and syphilis positive cases in the key population and general population

Categories		HIV +			Syphilis +		
		General Population	Key population	p value	General population	Key population	p value
Positive samples, n (%)		1,365	2,248		1,842	3,136	
Men, n (%)		874 (64)	2,211 (98)	<0.0001	1,285 (70)	3,048 (97)	<0.0001
Ethnicity, n (%)							
	White	917 (67)	1,612 (72)	0.0151	1,311 (71)	2,239 (71)	0.4647
	Not white	424 (31)	620 (27)		481 (26)	864 (27)	
	Not informed	24 (2)	16 (1)		50 (3)	33 (2)	
Median age (IQR) - years		36 (28-44)	27 (23-32)	<0.0001	34 (26-36)	27 (23-33)	<0.0001
Age group, years, n (%)							
	0-10	0	0	<0.0001	1 (0)	0	<0.0001
	11-20	65 (5)	215 (9.5)		109 (5.9)	269 (8.6)	
	21-30	384 (28)	1,354 (60.2)		614 (33.3)	1,846 (59)	
	31-40	449 (33)	498 (22.1)		462 (25.1)	702 (22.3)	
	41-50	286 (21)	135 (6)		320 (17.3)	231 (7.36)	
	51-60	140 (10)	40 (1.7)		201 (10.9)	74 (2.3)	
	61 years old or more	40 (3)	6 (0.2)		135 (7.3)	12 (0.38)	
	Not informed	1 (0)	0		0	2 (0.06)	
Education level, n (%)							
	Elementary school	386 (28)	123 (5.5)	<0.0001	371 (20.4)	147 (4.9)	<0.0001
	High school	624 (46)	1,002 (44.6)		843 (45.7)	1,303 (41.5)	
	University education	292 (21.4)	1,067 (47.4)		481 (26)	1,554 (49.5)	
	None	3 (0.2)	2 (0)		7 (0.3)	1 (0)	
	Not informed	60 (4.4)	54 (2.4)		140 (7.6)	131 (4)	
Single, n (%)							
	Yes	881 (64.5)	1,983 (88.2)	<0.0001	1,402 (76.1)	2,851 (91)	<0.0001
	No	445 (32.6)	229 (10.1)		372 (20.2)	215 (6.8)	
	Not informed	39 (2.9)	36 (1.6)		68 (3.7)	70 (2.2)	

IQR: interquartile range; HIV: human immunodeficiency virus.

A significant difference was observed in positive cases when comparing general and key populations, with high rates found in key populations, mainly for MSM.

**Figure 3:**
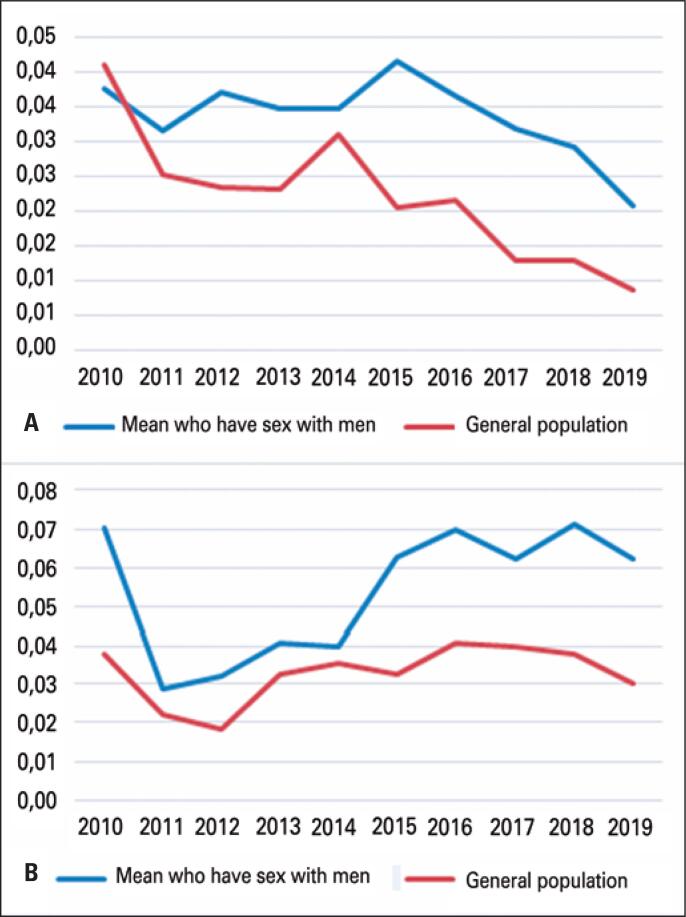
HIV (A) and syphilis (B) seroprevalence rates in the general population and in men who have sex with men

When comparing the prevalence of HIV between the general population and MSM, both have shown a decrease over the last few years, but the MSM group has a higher number of cases (Figure 3A). Increased syphilis prevalence in MSM is observed when compared to the general population (Figure 3B).

## DISCUSSION

The results of the present study show the prevalence rates of STI in a large population of subjects tested at the CTA during a 10-year period. The data showed high rates of HIV and syphilis positivity in key populations, emphasizing the need for public policies to assist these most vulnerable individuals.

The term key population refers to population groups that are more vulnerable to sexually transmitted diseases due to biological, social, or economic reasons. Consequently, they present a higher STI prevalence compared to the general population. Intravenous drug users, MSM, transgender, and sex workers are included in the key populations. Monitoring STDs in key populations is a reality in other countries. The information obtained on the incidence of STDs in these populations allows for prevention and health care measures, minimizing their impact on society.^(19)^

This study found a median prevalence of HIV of 5% among all tested individuals. However, when the key population was evaluated, the prevalence rate found was 11%, instead of 3% of the general population. These values are similar to previous reports in Brazil, in which the MSM group represents 14.2% of HIV positivity rates that characterizes the highest rate of virus contamination in the country, even when compared to other key populations, such as sex workers, whose prevalence is estimated at a rate of 5.3%, IDUs, at 9.9% and transgender, between 3.7% and 10.2%.^(12,14,20,21)^

Since 2013, increased testing has been conducted throughout the country with the objective to diagnose and provide early treatment to HIV-positive individuals within the 90-90-90 target recommended by WHO.^(2)^ This initiative in Brazil resulted in a progressive reduction in HIV rates. However, after 2014, an increase in syphilis positivity rates was detected, and currently, Brazil faces an epidemic of syphilis.^(22)^ These data highlight the importance of studying this disease and understanding its real impact to better intervene in its dissemination. The prevalence rate of syphilis found after 2016 stopped at around 10%. When we evaluated the positivity in the MSM group, the rate was at about 20%, higher than the previously reported 7.0%.^(23)^

Other risk groups also showed increased seroprevalence of syphilis, such as 8.5% in sex workers, 9.8% in IDUs, and 4.9 to 14.6% in transgender in epidemiological surveys conducted in Brazil.^(11,15,19)^ The epidemiological bulletin of 2018 reported 158,051 cases, showing an increase of 28.3% when compared to 2017.^(10,24)^

Furthermore, comparing the seroprevalence rate by age group, some differences were identified compared to the findings of other studies, such as a survey conducted in Turkey, where they reported a higher prevalence in people aged 36 to 45 years.^(25)^

On the other hand, in Louisiana and Massachusetts (USA), from 2012 to 2016, they reported a significant increase in syphilis cases in men aged between 20 and 44 years and highlight increased HIV and syphilis co-infection in the young population in these regions.^(26)^

In 2010, syphilis testing was primarily recommended in key populations. Thus, the increase in the number of cases may reflect higher testing and reporting, and might be more accurately assessed over the next few years.^(27)^ However, the same phenomenon also occurred in other countries in Latin America.^(28)^ One possible explanation is the change in the behavior of the populations, with an emphasis on the key populations, regarding the practice of anal sex without a condom, multiple sexual partners, drug use before sexual intercourse, and sexual encounters with anonymous people through the use of dating apps.^(12,21)^

The prevalence rates of HIV and syphilis observed in the general population group were 3% and 5%, respectively. These values are higher than expected for the population of Curitiba, which is usually 0.4% and 0.5%.^(13,23)^ It is important to note that the patients’ classification depended on their self-recorded status as part of the key population when filling out the form. When people did not fill out this section, they were grouped into the general population, so it is possible that some patients chose not to reveal the information.

When comparing the data with previous reports, there is a similarity with the predominance of men and white people. However, this profile is expected since the South of Brazil is primarily populated by European descendants, matching the description of patients tested at the CTA.

The present study also found a higher prevalence of HIV and syphilis in people with a higher level of education, which is probably due to the greater access of this population to testing, as well as to the identification of clinical manifestations, followed by medical assistance.

Concerning hepatitis serology, the low prevalence of HCV is probably due to the small number of intravenous drug (IVD) users in the studied population. In Curitiba, IVD users are referred to specialized clinics, and, thus, the direct search for testing by these individuals in the CTA is low, which impacts this analysis. Regarding HBV, vaccination against hepatitis B was deemed mandatory for children in 1998. In subsequent years, a national intensification of immunization programs contributed to decreasing this infection frequency rate.

In addition, actions aimed at key populations are a promising strategy for reducing the number of cases, to develop materials with languages accessible to the target audience, making forms of combined prevention understandable, which have shown promising results in places where they are used.^(29)^

This study has some limitations: - it was conducted with data from a single-center, and information is related only to people who were assisted at the CTA; therefore, the analysis and comparison of these data between populations must consider different profiles of the evaluated individuals; - since the researchers could not have access to patients’ identification, we were unable to evaluate the individuals who were tested repeatedly; and the key population was self-identified. However, this analysis is relevant because it includes many test results collected over a long period of time, allowing the identification of the most vulnerable and age groups with the highest prevalence of the investigated diseases.

## CONCLUSION

The HIV epidemic control has undergone several interventions over the years, and it is fundamentally based on extensive testing, early diagnosis, and universal treatment. The efficiency of these measures depends on identifying vulnerable key populations, for which should implement public policies that facilitate access to such actions. This study identified priority groups that would benefit from such interventions and confirmed the progress of a new epidemic of syphilis, which possibly reflects a reduction in the use of barrier protection methods. Thus, the broad approach to STIs among these individuals is a new challenge for the public health teams involved in assisting this population.
