# *Erratum:* HIV, syphilis, hepatitis B and C in key populations: results of a 10-year cross-sectional study, Southern Brazil

**DOI:** 10.31744/einstein_journal/2022AO6934E

**Published:** 2022-10-03

In the manuscript “HIV, syphilis, hepatitis B and C in key populations: results of a 10-year cross-sectional study, Southern Brazil”, DOI number: 10.31744/einstein_journal/2022AO6934, published at einstein (São Paulo). 2022;20:eAO6934, page 1, abstract results:

**Stated:**

A total of 67,448 samples were analyzed, 9,086 of these tested positive, 3,633 (56%) for HIV, 4,978 (77%) for syphilis, 340 (5%) for hepatitis C virus (HCV), and 135 (2%) for hepatitis B virus (HBV).

**It should be read:**

A total of 9,086 samples were positive across all samples tested, and yielded 3,633 (5%) for HIV, 4,978 (10%) for syphilis, 340 (1%) for hepatitis C virus (HCV), and 135 (<1%) for hepatitis B virus (HBV).

